# Linear and nonlinear ultrasound parameters attributed to anisotropy in granite

**DOI:** 10.1038/s41598-024-78367-6

**Published:** 2024-11-06

**Authors:** Seungo Baek, Kwang Yeom Kim, Gun Kim, Tae Sup Yun

**Affiliations:** 1 Department of Civil, Urban, Earth, and Environmental Engineering, Ulsan National Institute of Science and Technology (UNIST), Ulsan, 44919 Republic of Korea; 2 Department of Energy & Resources Engineering, Korea Maritime & Ocean University, Taejong-ro, Youngdo-gu, Busan, 49112 Republic of Korea; 3 School of Civil and Environmental Engineering, Yonsei University, Seoul, 03722 Republic of Korea

**Keywords:** Granite, Anisotropy, P-wave velocity, Attenuation, Acoustic nonlinearity parameter, Structural materials, Geology, Geophysics, Mineralogy, Petrology

## Abstract

The anisotropic nature of granite, a key factor affecting its mechanical properties, is inherently governed by its mineral alignment and the presence of orthogonal cleavage planes: rift, grain, and hardway. This study examines how these cleavage planes influence anisotropy, particularly in the context of microcracking formation and acoustic properties. A new measurement procedure for the acoustic nonlinearity parameter ($\:\beta\:$) is developed to address the well-known limitations of conventional linear ultrasound methods, including wave velocity and attenuation coefficient, in detecting microstructural changes induced by existing cleavage planes. Unlike other parameters, $\:\beta\:$ exhibits remarkable changes depending on the plane type, highlighting its high sensitivity to the mineral distribution in each cleavage plane and to the microcracks. A correlation between the linear and nonlinear parameters provides further evidence of the superiority of $\:\beta\:$ in detecting inherent microscale defects that develop in each plane and affect the anisotropic characteristics of granite. The findings of this study confirm that nonlinear ultrasound is capable of elucidating the mechanisms underlying the origin of anisotropy in granite due to microcracks, with broader implications for understanding unidentified chemical and mechanical phenomena in geological materials.

## Introduction

Rock anisotropy plays an instrumental role in determining the orientation of splitting when subjected to mechanical loading^1–3^. Herein, anisotropy refers to the directional dependence of physical properties in rocks, influencing their geomechanical and hydraulic behaviors^4^. Understanding these anisotropic features, e.g., mineral alignment and texture, is thus crucial for practical applications such as mining, petroleum storage, and foundation of structures. In this regard, granite is a notable rock type due to its mineral and fabric characteristics^5–7^. Depending on the spatial distribution and orientation of constituent minerals and microstructure, the mechanical properties of granite are uniquely determined, resulting in the formation of distinct cleavage planes (specifically, rift, grain, and hardway), where anisotropic characteristics are prominently observed.

Morphological and petrophysical studies have clarified the identification of orthogonally located cleavage planes in granites and the nature of each plane, such as the degradation of anisotropy or ease of splitting in the order of rift, grain, and hardway planes. To better analyze the anisotropy of granite and its effect on mechanical properties, extensive methods have been employed. Among others, ultrasonic wave velocity techniques have been widely utilized for assessing the geomechanical properties of granites in a nondestructive way^8–14^. The results from measured velocity agree well with the tensile strength obtained from conventional methods like the Brazilian test: the hardway is the strongest, while the rift is the weakest plane, demonstrating success in quantifying anisotropy on cleavage planes^15–17^. Despite the ability to nondestructively read the anisotropy, technical challenges remain in the velocity measurement. Specifically, while the use of high frequency is required to sensitively examine the anisotropic feature below a millimeter, it entails a highly scattering environment in which the propagating wave is significantly attenuated. Consequently, this leads to significant wave attenuation, resulting in a low signal-to-noise ratio (SNR) and insufficient sensitivity of velocity to the cleavage-induced anisotropy^18^.

The challenge of detection sensitivity in the wave velocity measurements can be effectively addressed by measuring the attenuation coefficient. This involves determining the loss of ultrasonic energy, which varies with the nominal frequency of the transducer; higher frequencies result in greater energy loss. By selecting an optimal frequency, one can precisely monitor local microstructural changes on cleavage planes^19,20^. However, attenuation measurement has the same scattering issue as velocity measurement because microstructures scatter high-frequency ultrasonic waves, while low-frequency waves lose their sensitivity to these microstructures. Additionally, the sensitivity of attenuation measurements to microstructures in granites needs further to be studied^21^. These technical limitations can restrict the effectiveness of velocity and attenuation measurements in revealing the anisotropy and its impact on the microstructures within granite cleavage planes.

More recently, nonlinear ultrasound and acoustic techniques have gained considerable attention owing to their ability to detect microstructural changes in porous materials^22–27^. These methods, including dynamic acousto-elastic testing^26–28^, nonlinear impact-based spectroscopy^29,30^, and second harmonic generation^24,31,32^, have been shown to be superior in detecting microscale defects. Consequently, they offer a more effective way to characterize a variety of physical behaviors in materials at the microscale. Nonetheless, these techniques have been less applied to identify anisotropy in granites.

This study presents a new measurement platform for consistently measuring both linear and nonlinear ultrasonic parameters to characterize the orthogonally distributed cleavage planes in granites. The experimental configuration proposed combines three different ultrasonic measurements: wave velocity, attenuation coefficient, and acoustic nonlinearity parameter. First, the measurement procedures for each parameter and the relevant underlying theory are described in detail. After the measurements of each parameter, the paper examines their sensitivity to each type of cleavage plane. Consequently, the paper discusses the relationship between the observed trends in measured parameters and the anisotropy present in granite samples.

## Materials and ultrasonic measurements

### Sample preparation

**Fig. 1:**
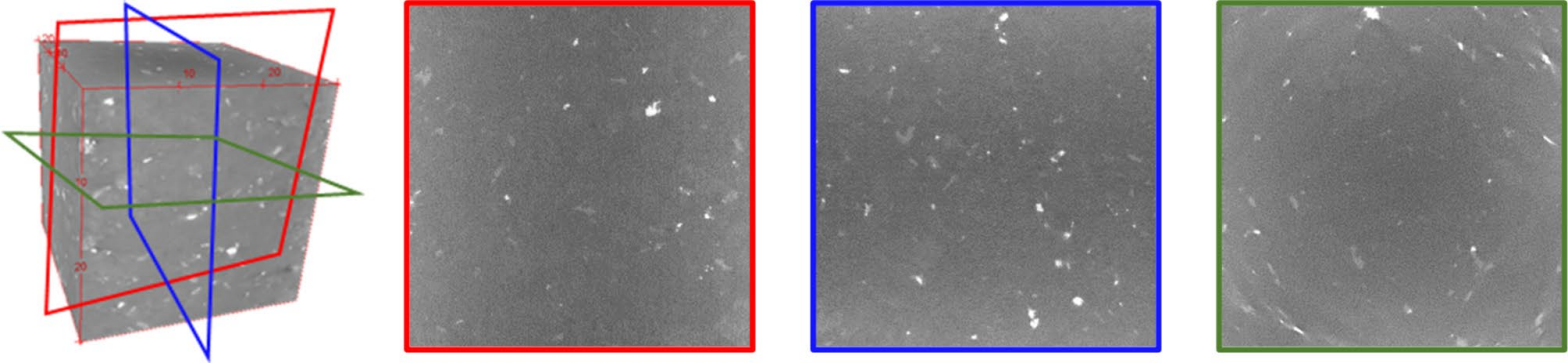
Examples of X-ray computed tomography images corresponding to each cleavage plane: Rift (red), Grain (blue), and Hardway (green).

The granite samples, formed in the Jurassic period, featuring gray color, were mined from the Pocheon quarry in South Korea. Note that the Pocheon granite, which intruded Precambrian gneisses, mainly consists of albite (35.9%), quartz (35.7%), microcline (25.8%), and biotite (2.6%). This Pocheon granite is characterized by three orthotropic cleavage planes, referred to as rift (average grain size of 0.94 mm), grain (1.01 mm), and hardway (0.91 mm)^4,6,7^. The orientation of each cleavage plane was determined during the quarrying process through field testing, which included visual inspection (e.g., visible fractures, microcracks, or mineral alignment) and small-scale fracture tests. Subsequently, the samples were cut along these cleavage planes, aligning with the faces of the cubic specimens. Two cubic specimens (50 × 50 × 50 mm^3^), named granite A and B, were prepared. Both specimens were intact and undamaged, with no visible defects on their surfaces. Figure 1 shows an example of the obtained X-ray computed tomographic images sliced parallel to the face of specimens, revealing the spatial complexity of the mineral facies. However, no distinct characteristics of the cleavage planes were visible in these images. It is important to note that while the presence of cleavage planes could be qualitatively identified through the statistical analysis of 3D X-ray images^33,34^, a comparable quantification of anisotropy was not feasible.

### Experimental setup for ultrasound measurements

**Fig. 2:**
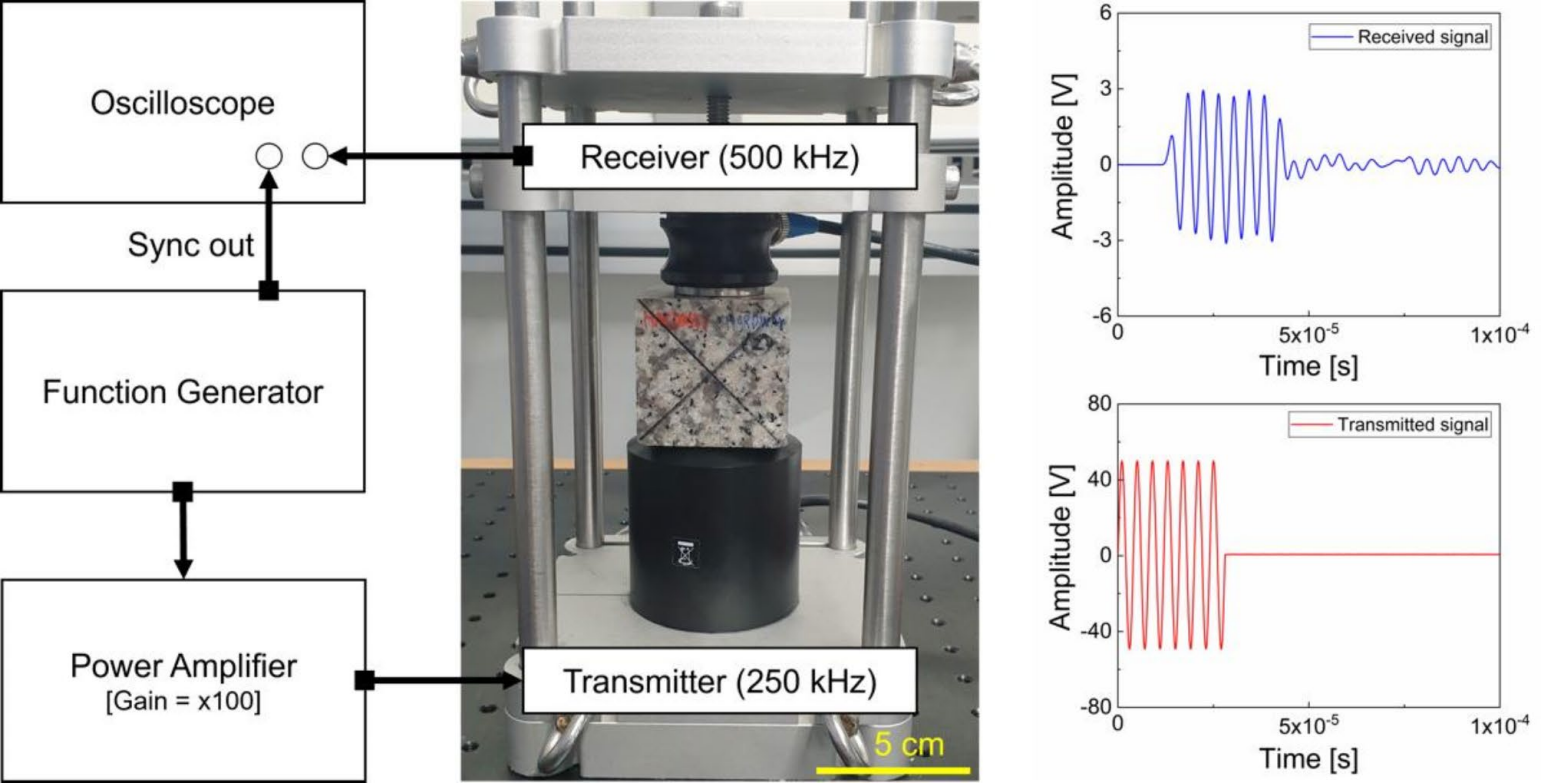
The experimental setup utilized for ultrasonic measurements: a 250 kHz transmitter and 500 kHz receiver were employed to estimate the values of $\:{c}_{P}$, $\:\alpha\:$, and $\:\beta\:$, respectively

Figure 2 shows the proposed experimental setup for systematically measuring three different ultrasonic parameters: wave velocity ($\:{c}_{P}$), attenuation coefficient ($\:\alpha\:$), and nonlinearity ($\:\beta\:$). A function generator (Keysight 33500B) was used to produce a tone-burst signal consisting of seven cycles with a pulse repetition frequency of 100 Hz. This voltage signal was amplified by 50 dB using a power amplifier (NF HAS 4052) and fed to the transmitter (transducer, Olympus V1012) with an excitation frequency of 250 kHz. The 250 kHz frequency was chosen to avoid interference from boundary-reflected waves in the forward-propagating waves (up to seven cycles). A 500 kHz receiver (transducer, Olympus V101) was used to capture the propagated longitudinal waves. As depicted in Fig. 2, a steel frame was constructed to precisely align the transducers with the sample, and a consistent amount of high vacuum grease (Dow Corning) was used to couple the sample with the transducers. It is important to note that the measurements of $\:{c}_{P}$, $\:\alpha\:$, and $\:\beta\:$ were performed under the same low contact pressure level to prevent any load-induced damage, such as creep from compression. The received signal was digitized and averaged 2,048 times to enhance the SNR using an oscilloscope (Keysight DSOX2024A). The entire setup was synchronized with a trigger signal from the function generator.

### Wave velocity (c_P_) and attenuation coefficient (α)

**Fig. 3:**
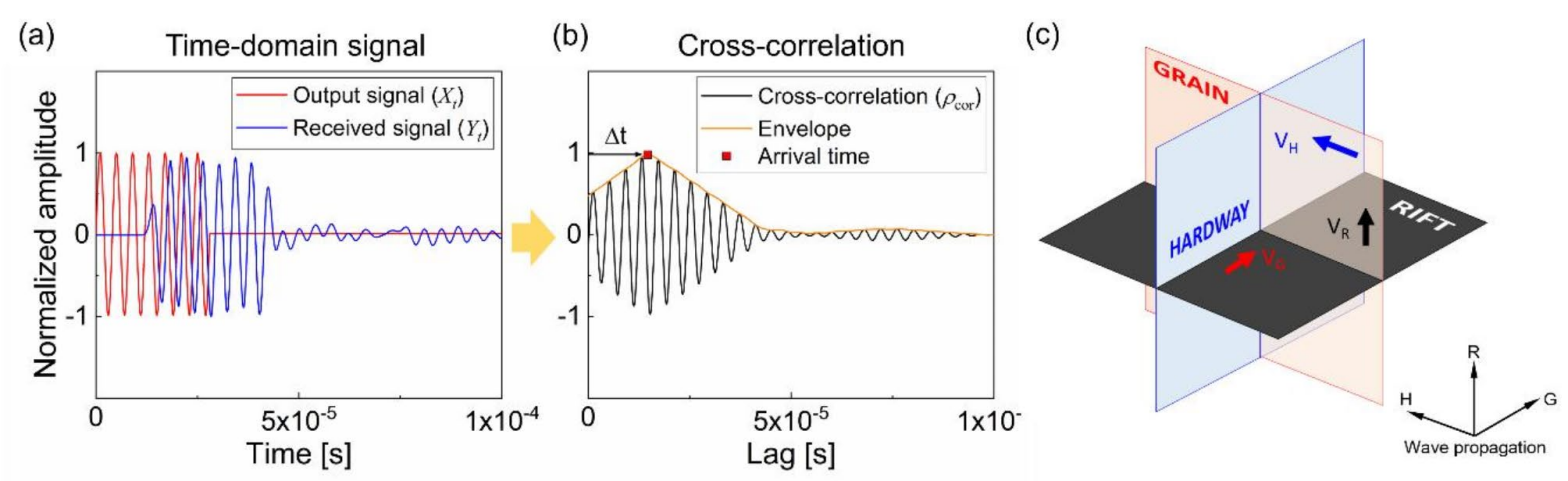
Determination of travel time for estimating P-wave velocity in granitic rock: (a) normalized transmitted (red) and received (blue) signals; (b) cross-correlated signal; and (c) wave propagation direction perpendicular to the cleavage plane.

Using the acquired signals, $\:{c}_{P}$ was determined via the cross-correlation method^33,35^. Figure 3a shows a normalized transmitted signal ($\:{X}_{t}$) in a reddish color and a received signal ($\:{Y}_{t}$) in a bluish color. The normalized cross-correlation ($\:{\rho\:}_{cor}$) between $\:{X}_{t}$ and $\:{Y}_{t}$ was calculated using a series of time lags ($\:k$), as expressed in Eq. (1). After applying the Hilbert transform to compute the envelope of $\:{\rho\:}_{cor}$, the time corresponding to the maximum point of the envelope was designated as the travel time ($\:\varDelta\:t$) (Fig. 3b).

1 $$\:{\rho\:}_{cor}\left(k\right)=cor\left({X}_{t+k},{Y}_{t}\right)=\frac{cov({X}_{t+k},{Y}_{t})}{\sqrt{var\left({X}_{t+k}\right)var\left({Y}_{t}\right)}}$$

where $\:cor$ represents the cross-correlation function, $\:cov$ the covariance function, and $\:var$ the variance function. The velocity was calculated using the thickness of the samples and the estimated $\:\varDelta\:t$. The accuracy of this method was confirmed in a previous study^9^. Figure 3c indicates that the targeted cleavage plane was oriented perpendicular to the wave propagation direction, and the $\:{c}_{P}$ measurement reflected the damage state of the corresponding cleavage plane. The $\:{c}_{P}$ measurement was repeated three times for each plane to achieve a statistically acceptable value.

**Fig. 4:**
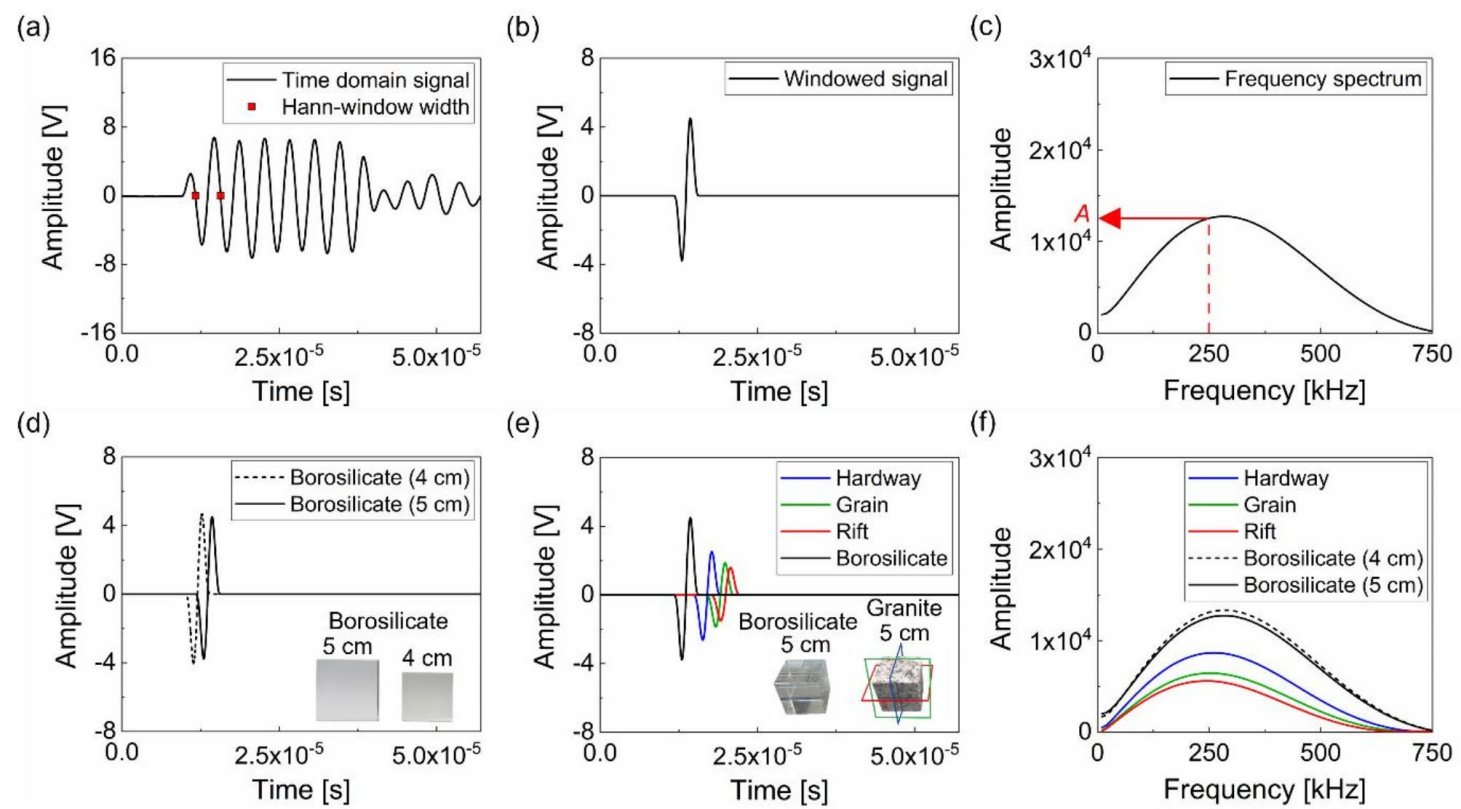
The proposed method for measuring attenuation. (**a**,**c**) Shows the selected windowed signal and its power spectrum. (**d**,**f**) Illustrates the changes in the magnitude of the spectrum for each cleavage plane and a pair of borosilicates. The absolute $\:\alpha\:$ value for borosilicate is first estimated then the value for granite is obtained by comparing the relative change of the amplitudes

The setup for measuring $\:\alpha\:$ was identical to that for velocity, except for the use of two borosilicate samples of different thicknesses (40 mm and 50 mm) as reference materials. Figure 4 details the procedure for measuring $\:\alpha\:$. The received 250 kHz-wave packet was obtained (Fig. 4a), and the first cycle of the received time-domain signal was cropped by a Hann window to minimize the coherent and incoherent noises (Fig. 4b). A fast Fourier transform (FFT) was then applied to the windowed signal to determine the amplitude ($\:A$) (Fig. 4c). As shown in Fig. 4d, the comparison of two amplitudes measured from 40 mm to 50 mm thickness allows for calculating absolute value of $\:\alpha\:$ for borosilicate sample, approximately 31.3 dB/m. This measured value of $\:\alpha\:$ is very close to the one introduced (30 ~ 90 dB/m) in the references^36,37^. Then, using the setup for 50 mm thickness, a relative comparison between borosilicate^38^ and granites was conducted to measure $\:\alpha\:$ for granite. Note that the measured $\:{c}_{P}$ and density ($\:\rho\:$) for borosilicate cubes were approximately 5400 m/s and 2.34 g/cm^3^, respectively (Fig. 4d). Compared to borosilicate, the first arrival time in the time domain for each granite cleavage plane differed (in the order of hardway, grain, and rift planes), as depicted in Fig. 4e. Similarly, the amplitude ($\:A$) in both time and frequency domains exhibited a decreasing trend following the same sequence, as illustrated in Fig. 4e and f.

As described in Eq. (2), the measured $\:{c}_{P}$ and $\:A$ were first applied to a complex diffraction correction function ($\:D$) to estimate α for borosilicate^39^.

2 $$\:D\left(f;x\right)=1-{e}^{-i\left(2\pi\:/s\right)}[{J}_{0}\left(2\pi\:/s\right)+i{J}_{1}(2\pi\:/s\left)\right]$$

where $\:{J}_{v}$ is the Bessel function of the first kind of order $\:v$, and $\:s=\left({c}_{P}\bullet\:x\right)/\left(f{a}^{2}\right)$ with the propagation distance $\:x$, frequency $\:f$, and radius of transducer $\:a$. The attenuation coefficient for the borosilicate can be obtained in the following form:

3 $$\:{\alpha\:}_{borosilicate}\left(f\right)=\:\frac{20}{{L}_{2}-{L}_{1}}\left[\text{log}\left(\frac{A(f;{L}_{1})}{A(f;{L}_{2})}\right)-\text{log}\left(\frac{D(f;{L}_{1})}{D(f;{L}_{2})}\right)\right]\:$$

where $\:{L}_{1}$, and $\:{L}_{2}$ indicate the length of each borosilicate cube. The estimated $\:\alpha\:$ for borosilicate was about 31.3 dB/m with a standard deviation of 3.24 dB/m from Eqs. 2 and 3. To determine $\:\alpha\:$ for the granitic samples, the amplitudes in the frequency domain (Fig. 4f) for 50 mm cubic borosilicate ($\:{A}_{borosilicate}$) and granite ($\:{A}_{granite}$) were compared using Eq. (4):

4 $$\:{\alpha\:}_{granite}\left(f\right)=\frac{20}{{L}_{2}}\left[\text{log}\left(\frac{{A}_{borosilicate}\left(f;{L}_{2}\right)/{T}_{borosilicate}}{{A}_{granite}\left(f;{L}_{2}\right)/{T}_{granite}}\right)\right]+{\alpha\:}_{borosilicate}\left(f\right)\:$$

Here, $\:T$ represents the transmission coefficient used to correct for energy losses due to acoustic impedance ($\:Z={c}_{P}\rho\:$) mismatching between the transducer and the testing material. Theoretically, $\:T$ can be calculated in the following form:

5 $$\:{T}_{material}=\frac{4{Z}_{material}{Z}_{transducer}}{{\left({Z}_{material}+{Z}_{transducer}\right)}^{2}}$$

The value of the $\:{Z}_{transducer}$ is determined based on the properties of the PZT which is raw materials of transducer^40^. Equation (4) and Fig. 4 indicate that $\:{\alpha\:}_{granite}$ is significantly greater than $\:{\alpha\:}_{borosilicate}$, as the logarithmic ratio of $\:{A}_{borosilicate}$ to $\:{A}_{granite}$ is less than 1.

It is crucial to note that granite is a heterogeneous material that causes a significant scattering environment, where the amplitude of reflected echo signals would inevitably be low. Therefore, traditional methods such as the pulse-echo method^41^, which rely on the use of reflected echo signals, are not suitable for assessing anisotropy in granite due to low SNR. Nonetheless, this proposed method effectively estimates $\:\alpha\:$ for granite by consistently using the first-arrived signals with a high SNR, followed by diffraction correction, thereby providing an absolute value of $\:\alpha\:$ (dB/m).

### Acoustic nonlinearity parameter (β)

The second harmonic generation (SHG) technique was used to estimate $\:\beta\:$ in propagating P-waves^23,24,31^. The exact expression of $\:\beta\:$ can be derived by solving one-dimensional nonlinear wave equation that combines the nonlinear constitutive equation and the equation of motion^42,43^, as shown in Eq. (5):

6 $$\:\frac{{\partial\:}^{2}u}{\partial\:{t}^{2}}={c}_{P}^{2}\left[1-\beta\:\frac{\partial\:u}{\partial\:x}\right]\frac{{\partial\:}^{2}u}{\partial\:{x}^{2}}$$

where $\:u$ is the particle displacement. The harmonic solution of this nonlinear wave equation is expressed as:

7 $$\:u={A}_{1}\text{cos}\left(kx-\omega\:t\right)-\frac{\beta\:x{k}^{2}{A}_{1}^{2}}{8}\text{cos}(2kx-2\omega\:t)$$

Equation (7) gives the relationship between the measured amplitude of fundamental (*ω*) and second (2*ω*) harmonics, the wave number, $\:k$, and *β* :

8 $$\:\beta\:=\frac{8\left|{A}_{2}\right|}{x{k}^{2}{A}_{1}^{2}}\equiv\:\frac{{A}_{2}}{{A}_{1}^{2}}$$

**Fig. 5:**
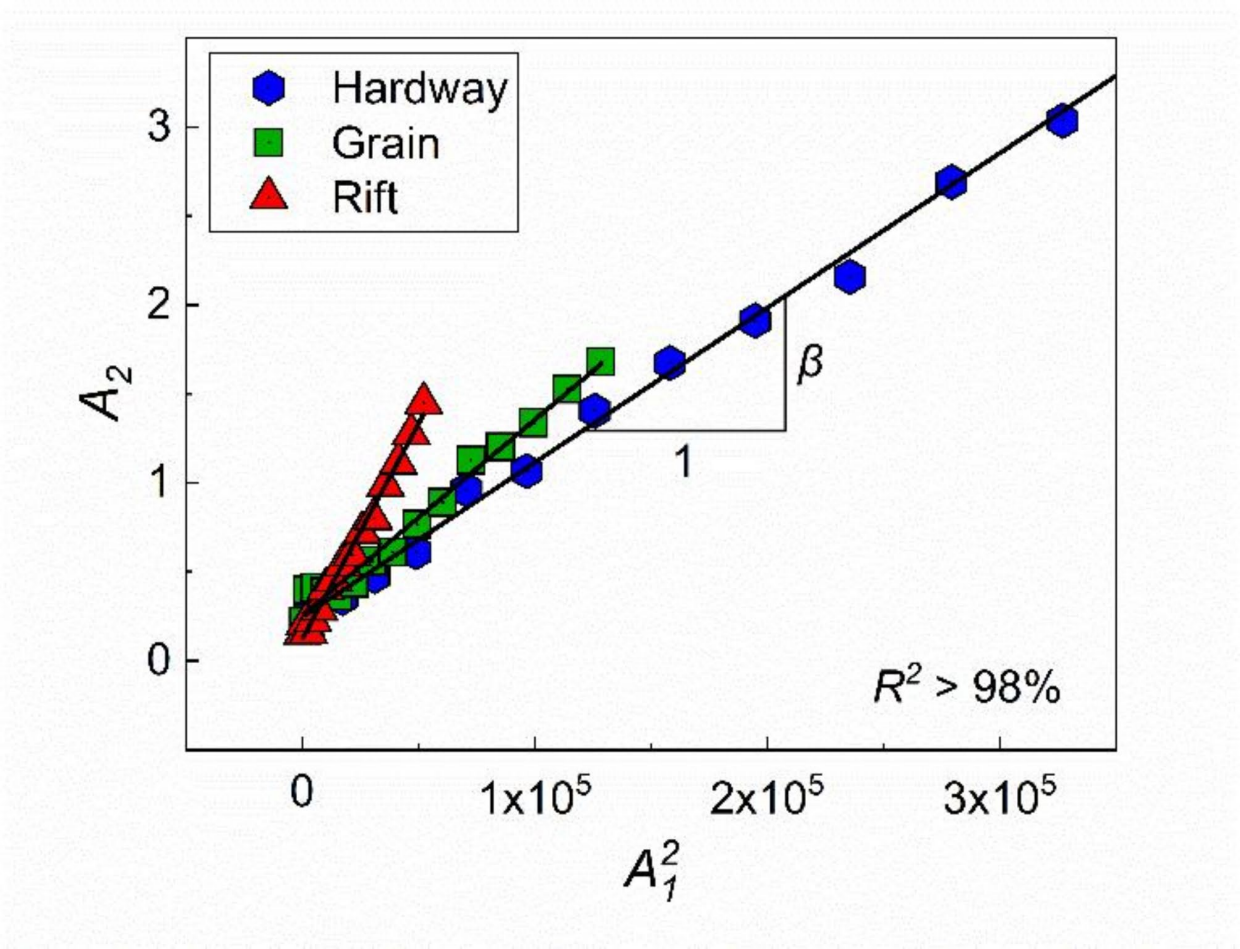
Ratio of $\:{A}_{2}$ to $\:{A}_{1}^{2}$ for each cleavage plane in granite A. The slope of the linear fit yields $\:\beta\:$, and all linear regressions for the estimated points exhibit $\:{R}^{2}$ values higher than 98%

As the excitation frequency and propagation distance were fixed to 250 kHz and 50 mm, $\:\beta\:$ can be written as a direct function of $\:{A}_{1}$ and $\:{A}_{2}$, which is the slope of linear fit for the measured $\:{A}_{1}^{2}$ versus $\:{A}_{2}$^32,44^, as shown in Fig. 5.

**Fig. 6:**
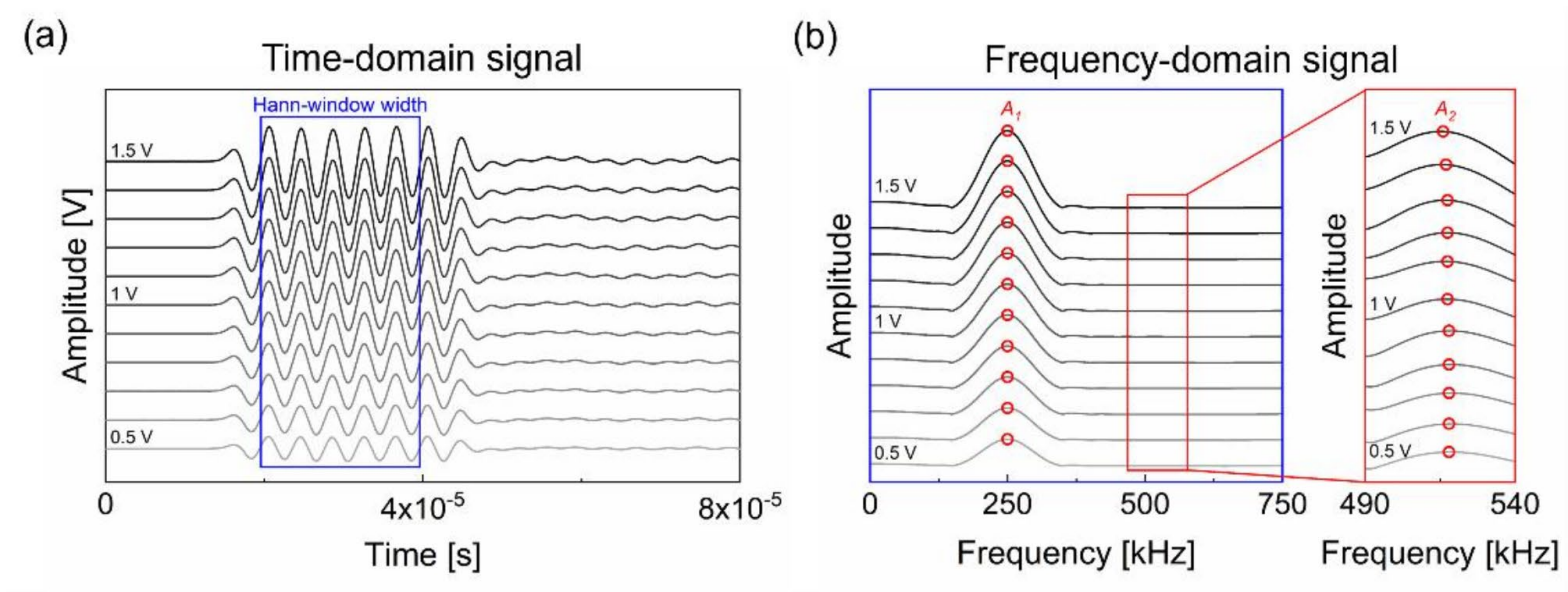
Example of obtained time-domain signal and frequency spectrum. (**a**) Shows the steady-state part of received signals with the increasing input voltage, and (**b**) shows the trend of $\:{A}_{1}$ and $\:{A}_{2}$ with voltage level, confirming a monotonically increasing magnitude with increasing voltage.

To measure $\:\beta\:$, a series of the transmitted signals obtained with varying input voltage from 500 mV to 1.5 V with an interval of 100 mV was used. Specifically, as shown in Fig. 6a, the five cycles of the received signal in the steady-state part were used for the FFT. Note that the use of five cycles was determined based on the estimation of the first arrival time of the boundary-reflected waves in the granite samples. This estimation ensured not only the elimination of the geometry-related noise but also the achievement of a high SNR for a precise measurement of $\:\beta\:$. Figure 6b shows the determined $\:{A}_{1}$ and $\:{A}_{2}$ for each input voltage. The slope of linear fit for the measured $\:{A}_{1}^{2}$ versus $\:{A}_{2}$ then became $\:\beta\:$, as per Eq. (7). In this study, the determination coefficient ($\:{R}^{2}$) for the linear fit, exceeded 98% in all cases. Figure 5 shows an example of the measured $\:\beta\:$ for each cleavage plane of granite A. The obtained $\:\beta\:$ was then used to assess the anisotropy originating from each cleavage plane.

## Results and discussion

### Characterization of mechanical properties using c_P_ and α

**Fig. 7:**
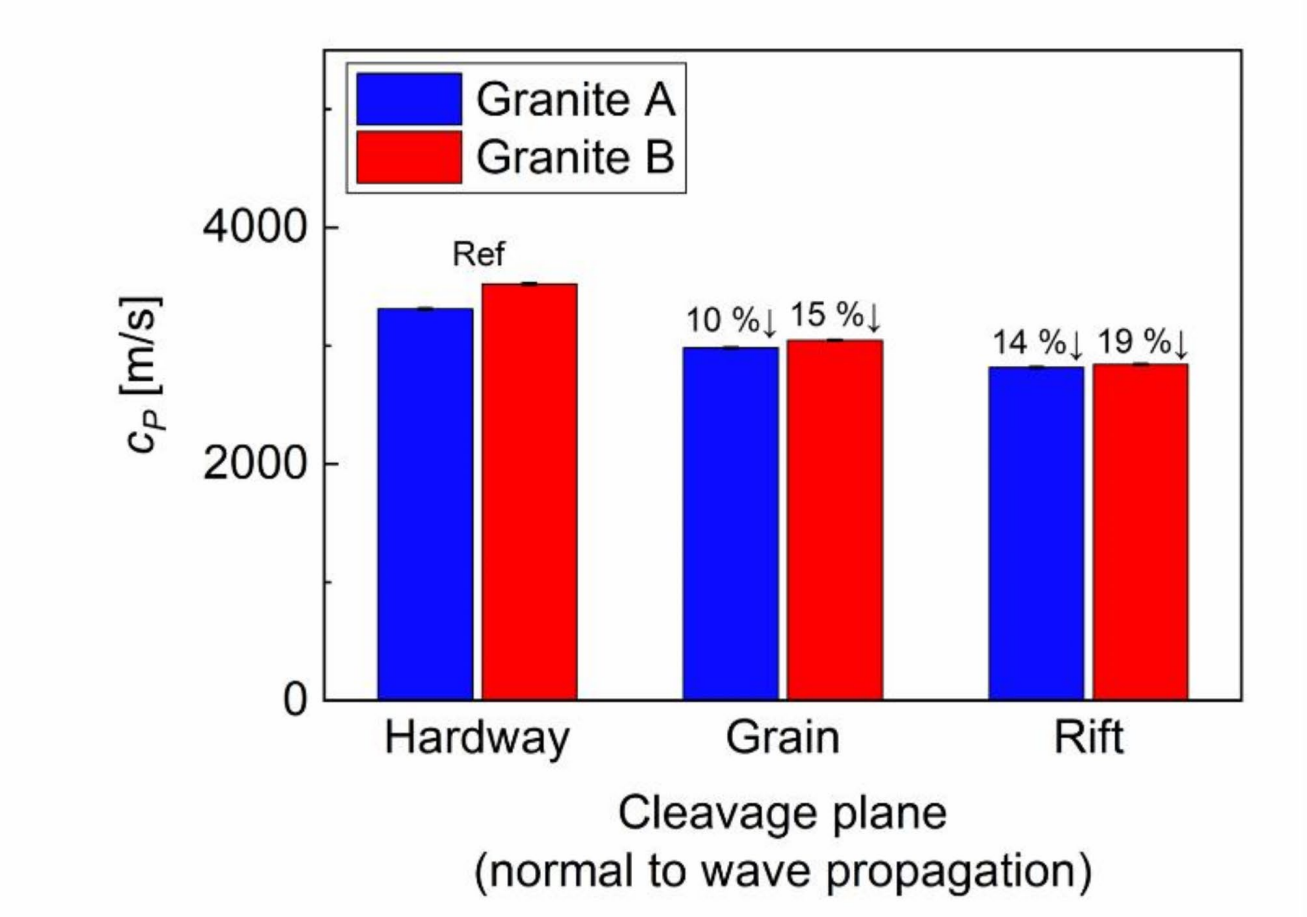
Estimated values of wave velocities for granitic samples.

Figure 7 presents the measured $\:{c}_{P}$ for each specimen. Note that the results were averaged from three repetitions with their standard deviation. The averaged velocity values range from 2,817 m/s to 3,311 m/s for granite A and 2,843 m/s to 3,524 m/s for granite B. These are in close agreement with values from the references^9,19^ and the conventional time-of-flight method (i.e., peak-to-peak method) shown in Table 1. Notably, the lowest $\:{c}_{P}$ values occurred when the rift plane was aligned normal to the wave propagation direction. In contrast, the highest $\:{c}_{P}$ was observed when the hardway plane was oriented normal to the wave propagation direction: 3,311 m/s for granite A; and 3,524 m/s for granite B. As wave velocity is directly linked to the internal constituents and their distribution, the variance in $\:{c}_{P}$ values provides an important evidence that the rift is the weakest cleavage plane and the hardway is the most intact cleavage plane. Moreover, the reduction in $\:{c}_{P}$ from hardway to rift planes (around 14% fr granite A and 19% fr granite B) validates a systematic detection of anisotropy through $\:{c}_{P}$. Despite this capability to identify the cleavage plane, distinguishing anisotropic variations between granite A and B samples was challenging, as shown in Table 1. This is because of the low sensitivity of $\:{c}_{P}$ to anisotropic features, particularly at the microscale. Previous studies^18,23,44^ indicate that the sensitivity of $\:{c}_{P}$ was not enough to characterize the material state below the mesoscale, and thus not sufficient for precise assessment of microstructural change in the porous medium.

**Fig. 8:**
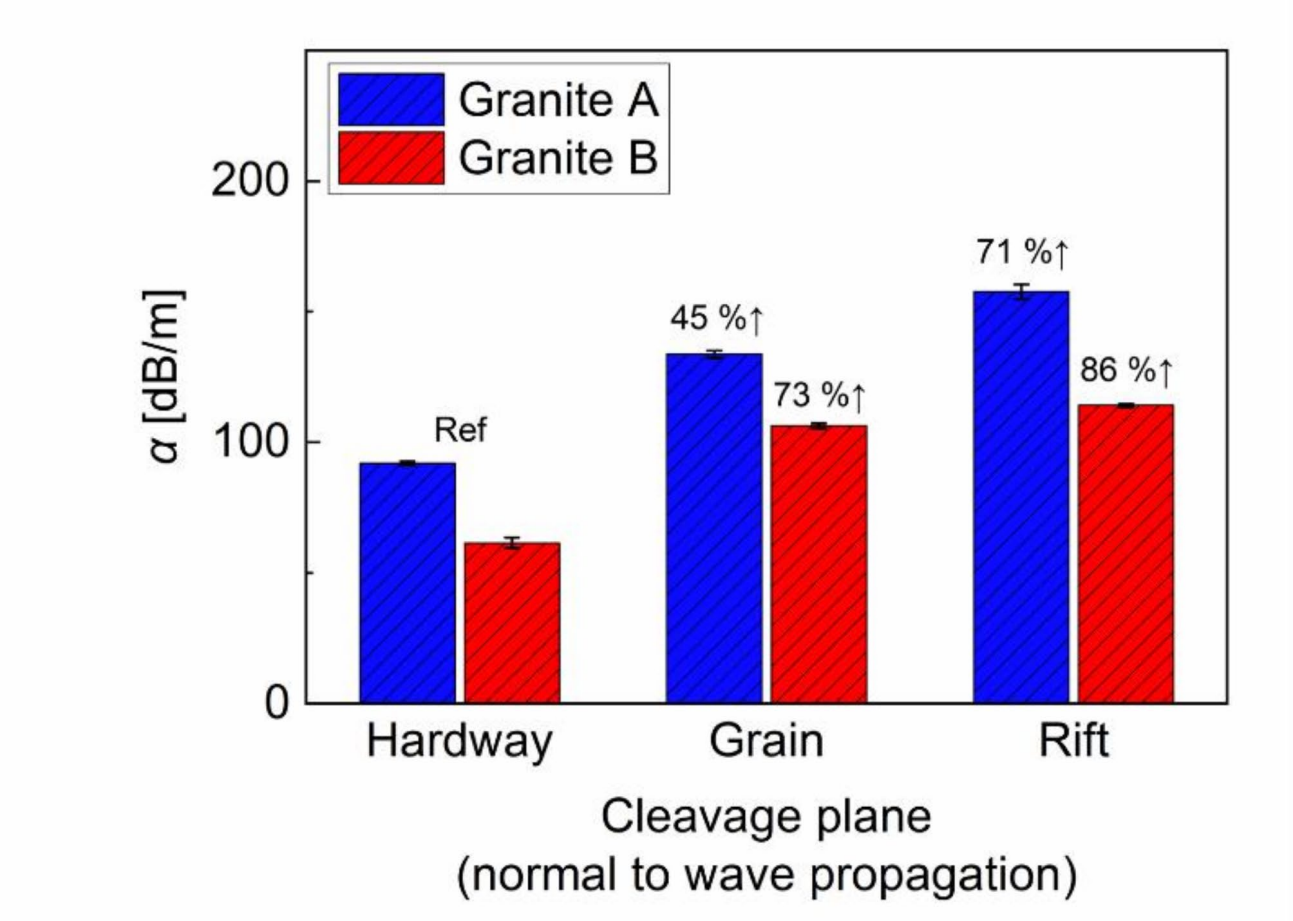
Estimated values of the attenuation coefficient with corrections for diffraction and transmission loss.

The original signals received from granite and borosilicate were corrected for the diffraction effect, and then using Eq. (4), $\:\alpha\:$ was computed for three cleavage planes. Table 2 summarizes the measured $\:\alpha\:$ for each cleavage plane, both with and without the application of diffraction and transmission loss correction. The results indicate that conventional methods^21^ lead to overestimating $\:\alpha\:$, causing a measurement discrepancy that approximately ranges between 23% and 31%. This overestimation is attributed to transmission losses due to impedance mismatching and the spreading of the ultrasound beam as it propagates through the material. Nonetheless, the proposed method provides the procedure of transmission loss and diffraction correction, enabling the accurate measurement of $\:\alpha\:$ and thereby facilitating a more previse assessment of the cleavage effect. As shown in Fig. 8, the rift plane exhibited the highest $\:\alpha\:$ value, approximately 157.57 dB/m for granite A and 114.11 dB/m for granite B, while the lowest $\:\alpha\:$ appeared in the hardway plane (91.93 dB/m for granite A, and 61.65 dB/m for granite B). Note that the increment between each cleavage plane was approximately 45% for hardway-to-grain and 71% for hardway-to-rift in granite A, and 73% for hardway-to-grain and 86% for hardway-to-rift in granite B. This observation well supports that the rift plane inherently contains more microscale defects than the grain and hardway planes. Unlike $\:{c}_{P}$, the trend of $\:\alpha\:$ for each cleavage plane was reversed, which coincided with other methods for $\:\alpha\:$ introduced in previous studies^45–48^. Furthermore, the variation in $\:\alpha\:$ was more pronounced than that in $\:{c}_{P}$, highlighting its sensitivity to microstructural change and other sound matrices along cleavage planes. In a scattering environment caused with 250 kHz, the intrinsic defects in the cleavage planes resulted in a significant loss of ultrasonic energy than the reduction in $\:{c}_{P}$, demonstrating higher sensitivity of $\:\alpha\:$ than $\:{c}_{P}$. Owing to the high sensitivity, $\:\alpha\:$ could discern the anisotropy variation between samples, a feature not evident in the $\:{c}_{P}$ measurements. Figure 8 shows granite A exhibiting higher $\:\alpha\:$ value across all cleavage planes than granite B, demonstrating that more significant anisotropy and inherent defects were developed in granite A, despite both granite samples originating from the same quarry. Table 2 summarizes the measured attenuation coefficients for each cleavage plane.

**Table 1:** Averaged P-wave velocities (m/s) for granite samples.

Cleavage plane	Granite A		Granite B	
	Peak-to-peak	Cross-correlation	Peak-to-peak	Cross-correlation
Hardway	3,714.5 ($\:\pm\:$4.38)	3,310.8 ($\:\pm\:$5.27)	3,898.6 ($\:\pm\:$6.29)	3,523.9 ($\:\pm\:$5.64)
Grain	3,226.5 ($\:\pm\:$6.83)	2,982.4 ($\:\pm\:$4.13)	3,263.8 ($\:\pm\:$7.01)	3,046.4 ($\:\pm\:$4.22)
Rift	2,989.2 ($\:\pm\:$6.08)	2,817.0 ($\:\pm\:$3.80)	2,999.8 ($\:\pm\:$6.29)	2,843.1 ($\:\pm\:$3.88)

**Table 2:** Averaged attenuation coefficients (dB/m) for granite samples with and without correction.

Cleavage plane	Granite A		Granite B	
	Uncorrected	Corrected	Uncorrected	Corrected
Hardway	118.95 ($\:\pm\:$0.77)	91.93 ($\:\pm\:$0.77)	83.87 ($\:\pm\:$2.08)	61.51 ($\:\pm\:$2.08)
Grain	175.17 ($\:\pm\:$1.37)	133.74 ($\:\pm\:$1.37)	153.91 ($\:\pm\:$0.98)	106.31 ($\:\pm\:$0.98)
Rift	206.80 ($\:\pm\:$2.83)	157.57 ($\:\pm\:$2.83)	159.22 ($\:\pm\:$0.57)	114.11 ($\:\pm\:$0.57)

**Table 3:** Averaged acoustic nonlinearity parameter (unitless) for granite samples.

Cleavage plane	Granite A	Granite B
Hardway	1.34 × 10^− 5^ ($\:\pm\:$6.28$\:\times\:$10^−7^)	8.47 × 10^− 6^ ($\:\pm\:$2.86$\:\times\:$10^−7^)
Grain	2.05 × 10^− 5^ ($\:\pm\:$8.14$\:\times\:$10^−7^)	1.20 × 10^− 5^ ($\:\pm\:$3.43$\:\times\:$10^−7^)
Rift	4.37 × 10^− 5^ ($\:\pm\:$2.34$\:\times\:$10^−6^)	2.25 × 10^− 5^ ($\:\pm\:$5.83$\:\times\:$10^−7^)

### Characterization of microcracks using acoustic nonlinearity parameter β

**Fig. 9:**
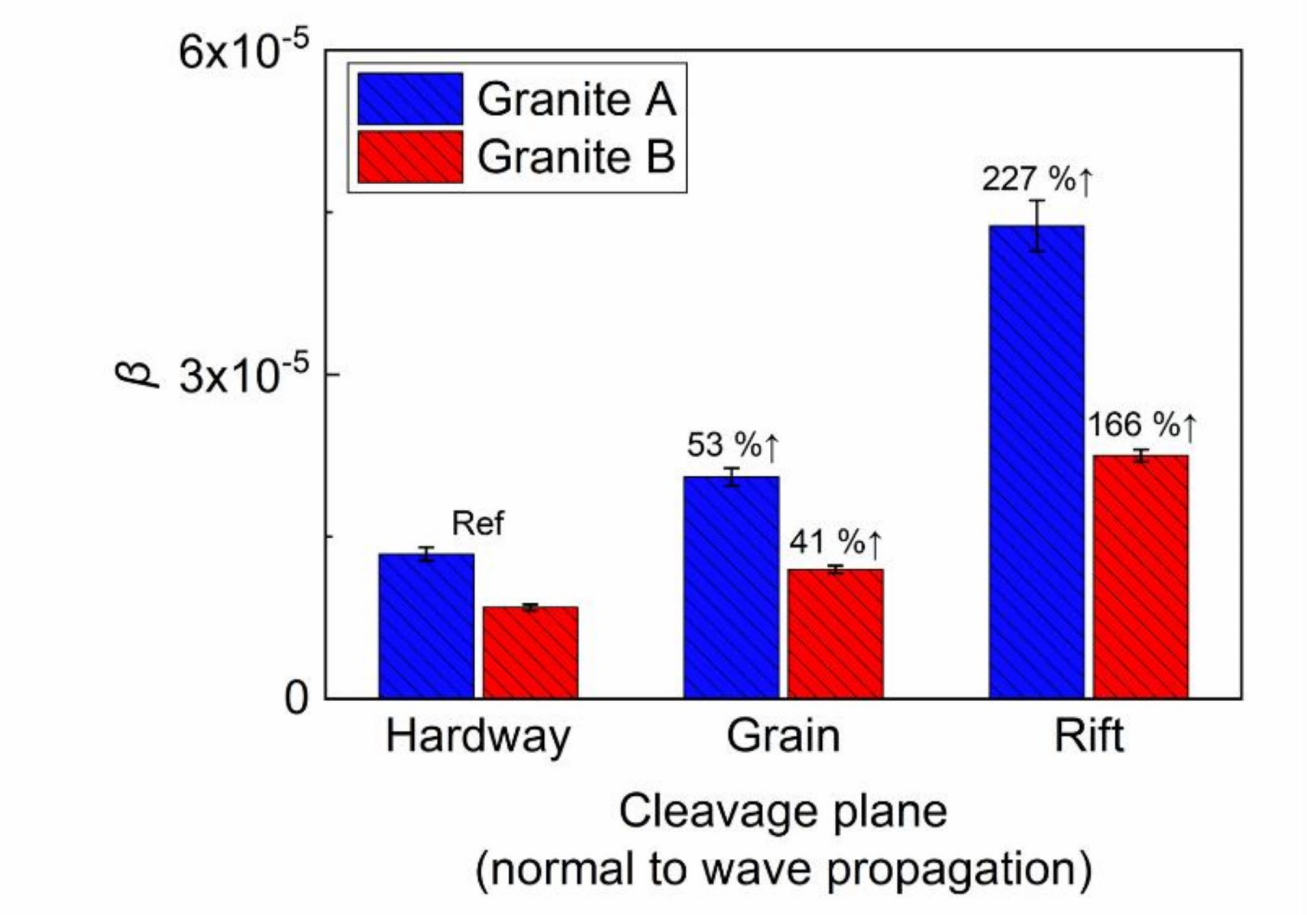
Estimated values of acoustic nonlinearity parameter.

Although the SHG technique has been applied to various substances in several studies^32,49^, this paper makes an original contribution by employing the SHG technique to measure $\:\beta\:$ for the first time, specifically to assess the anisotropy in granites induced by cleavage planes. The main focus is on identifying defects and structural changes at the microscale on these cleavage planes. Table 3 summarizes the $\:\beta\:$ values obtained for all cleavage planes. Note that the $\:\beta\:$ value of borosilicate, a material known for its high linearity, was measured at 5.08 × 10^−6^. This is much lower than the $\:\beta\:$ value of granite, confirming the high inherent nonlinearity of granite. For granite A, $\:\beta\:$ increases by 53% from the hardway-to-grain plane and 227% from the hardway-to-rift plane, as illustrated in Fig. 9. This noticeable increase in $\:\beta\:$ highlights its greater sensitivity to cleavage-induced anisotropy compared to other parameters like $\:{c}_{P}$ and $\:\alpha\:$. Such anisotropy, as evidenced by $\:\beta\:$, enables a clear distinction both between types of cleavage and among the samples. Figure 9 further demonstrates that $\:\beta\:$ measured for granite A is considerably higher than that for granite B, confirming a more pronounced development of anisotropy and material nonlinearity in granite A, despite the same origin. Additionally, $\:\beta\:$ facilitates distinguishing between the grain and rift planes in granite B. Such differentiation was not achieved based on the $\:\alpha\:$ measurements due to its low sensitivity to microstructural variations. The outcomes of the nonlinearity measurements highlight the sensitivity of $\:\beta\:$ to various aspects of the anisotropic nature of cleavage planes, such as mineral constituents, geometrical configuration, and invisible defects^49^.

**Fig. 10:**
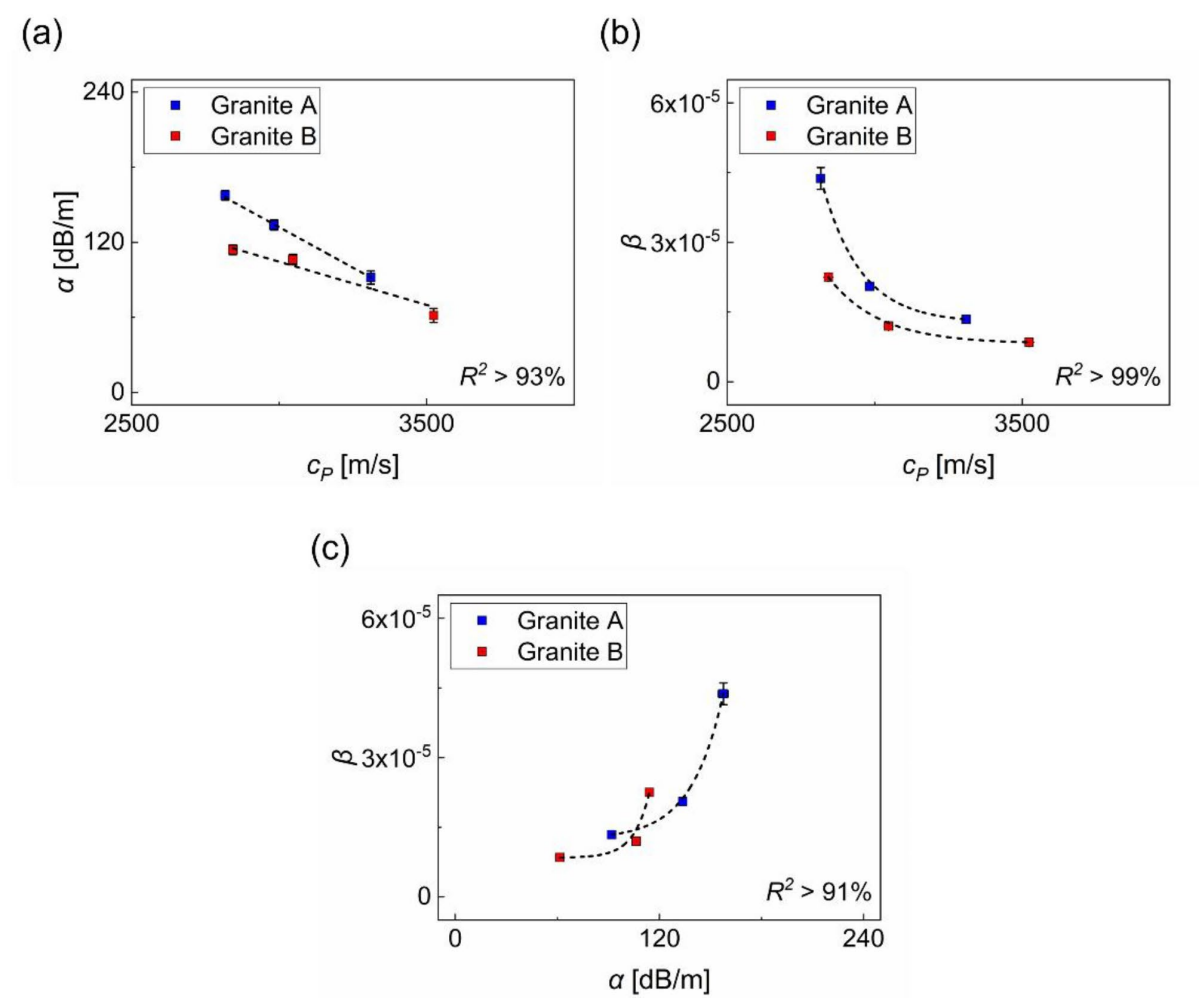
Comparison of three ultrasonic parameters: (**a**) wave velocity vs. attenuation (linear); (**b**) wave velocity vs. acoustic nonlinearity parameter (nonlinear); and (**c**) attenuation vs. acoustic nonlinearity parameter (nonlinear).

Figure 10 shows a correlation analysis for a direct comparison of three ultrasonic parameters: $\:{c}_{P}$, $\:\alpha\:$, and $\:\beta\:$. The relationship between $\:{c}_{P}$ and $\:\alpha\:$ exhibits a linear trend (with $\:{R}^{2}$ = 99% for granite A and 93% for granite B). In contrast, the correlations between $\:{c}_{P}$ and $\:\beta\:$, as well as $\:\alpha\:$ and $\:\beta\:$, follow a nonlinear curve characterized by exponential decay ($\:{R}^{2}$ = 99% for both granites A and B) and growth functions ($\:{R}^{2}$ = 99% for granites A and 91% for granite B), respectively. These results confirm the superior sensitivity of $\:\beta\:$ to the cleavage-induced anisotropy in granites compared to the other two linear parameters. The results observed in this study demonstrate the effectiveness of the proposed ultrasonic setup, which uses frequencies of 250 and 500 kHz to systematically and quantitatively measure three independent ultrasonic parameters. This approach successfully reveals the anisotropy present in the cleavage planes.

### Spatial scanning of mechanical properties

**Fig. 11:**
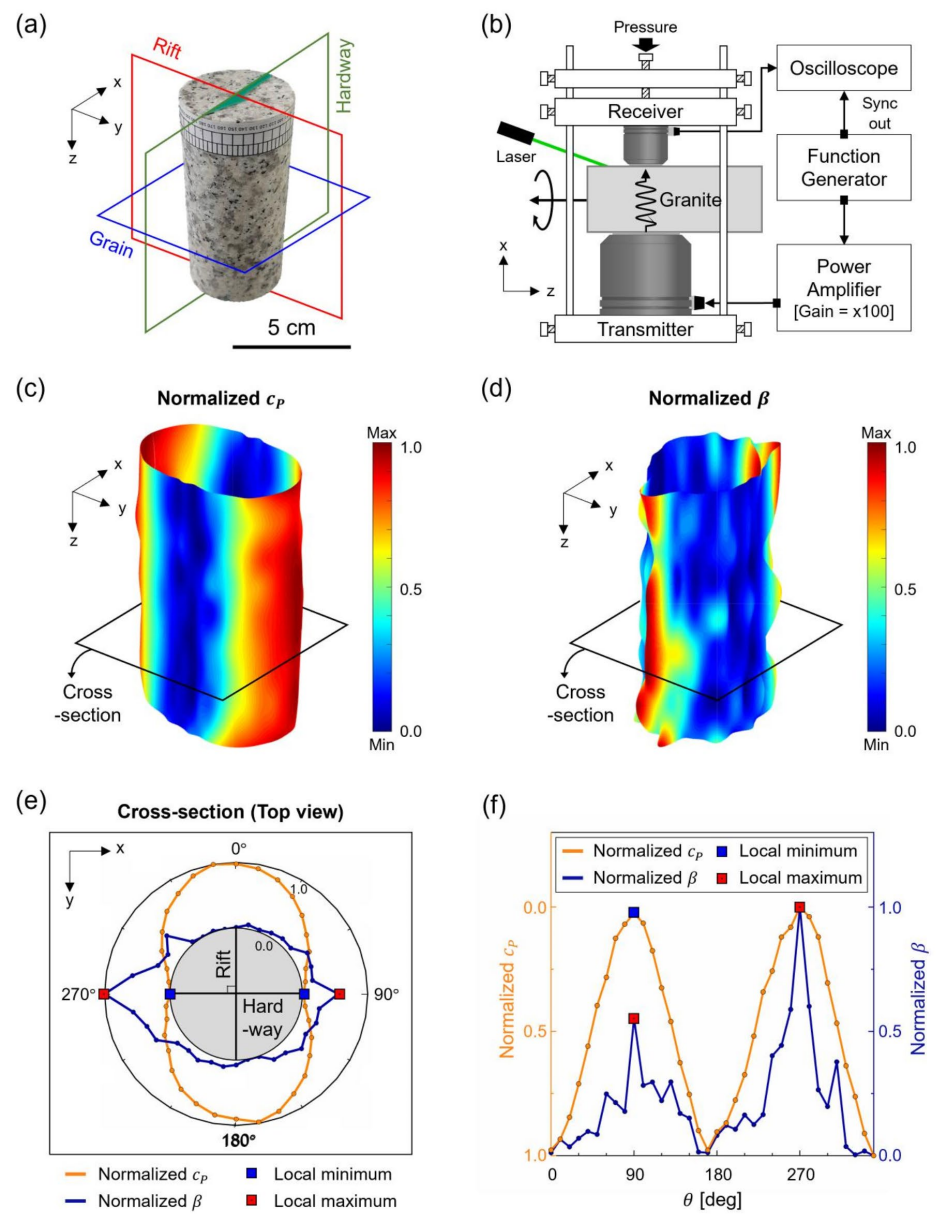
Spatial scanning of a cylindrical granite sample; (**a**) cleavage plane orientations within the cylindrical granite sample; (**b**) schematic of the ultrasonic scanning setup; (**c**) spatial distribution of normalized $\:{c}_{p}$; (d) spatial distribution of normalized $\:\beta\:$; (e) determination of the cleavage planes using the obtained cross-sectional image; and (f) comparison of directional variations in $\:{c}_{p}$ and $\:\beta\:$ within the cross-sectional image.

For a deeper understanding of the spatial distribution of microcracks and their effect on the anisotropic nature of granite, continuous scanning of both linear ($\:{c}_{p}$) and nonlinear ($\:\beta\:$) ultrasonic parameters was conducted. For the experiment, a cylindrical granite sample with a diameter of 5 cm and a height of 11 cm was prepared. The sample was cored from the Pocheon quarry in an identical way to the cubic samples. As shown in Fig. 11a, the grain plane was oriented parallel to the top and bottom surfaces while it maintained the orthogonality with the rift and hardway planes. For ultrasonic measurements, the setup introduced in Section “Experimental setup for ultrasound measurements” was used to consistently measure $\:{c}_{p}$ and $\:\beta\:$. Spatial scanning, as illustrated in Fig. 11b, was performed by adjusting the position of the sample at 10-degree intervals circumferentially and at 0.8 mm axially. Before mounting the transducers, the entire surface of the sample was covered with high vacuum grease. For each measurement point, the transducer was remounted with consistent low contact pressure to ensure stable contact conditions. A laser was used to precisely guide the scanning position during both the rotation and translation of the sample. Linear interpolation was applied to the measured $\:{c}_{p}$ and $\:\beta\:$ values to enhance spatial resolution^50,51^. The interpolated values were then rescaled using min-max normalization. As shown in Fig. 11c,d, the normalized parameters are visualized, showing different trends due to the detection sensitivity of each parameter. Finally, the circumferential distribution of both parameters was obtained with the polar coordinate plots (Fig. 11e), and their spatial variance was displayed in Fig. 11f.

Specifically, the images in Fig. 11e and f compare the variation of both parameters – $\:{c}_{p}$ (orange line) and $\:\beta\:$ (blue line) – measured along the plane marked by the black solid line in Fig. 11c and d. Here, the orientation of the cleavage plane is determined by connecting the local peak values of both parameters. As discussed in Section “Characterization of mechanical properties using c_p_ and α” and Characterization of microcracks using acoustic nonlinearity parameter β”, $\:{c}_{p}$ reaches its minimum while $\:\beta\:$ attains its maximum when the wave propagates normal to the rift plane. Therefore, in Fig. 11e, the orientation of the rift plane is perpendicular to the line connecting the local minimum values of $\:{c}_{p}$ or local maximum values of $\:\beta\:$. Consequently, the hardway plane is located along the perpendicular direction to the rift plane. This cross-sectional image in Fig. 11e confirms that the orientation of cleavage planes can be identified using both parameters, with the locations of maximum and minimum peaks nearly matching. However, as shown in Fig. 11f, both parameters exhibit different contrasts in their directional variation. While $\:{c}_{p}$ shows broad, symmetric lobes around local maximum and minimum values with small variation, $\:\beta\:$ pinpoints them within narrow, asymmetric lobes, making a clear distinction of the cleavage planes. This is due to the pronounced detection sensitivity of $\:\beta\:$ compared to $\:{c}_{p}$ for small-scale defects, allowing for more precise quantification of microstructural changes in granite, as discussed in Section “Characterization of microcracks using acoustic nonlinearity parameter β”.

Along the axis where the local peak values appear, i.e., the line connecting 90° and 270°, no significant spatial variation of $\:{c}_{p}$ was observed: 2613 m/s for 90°; and 2535 m/s for 270°, approximately 2.9% change. This verifies the similar elastic properties along the cleavage planes, regardless of the wave incident direction. Nonetheless, the asymmetric distribution of $\:\beta\:$ shows different values at each angle: 7.61 × 10^−5^ for 90°; and 10.28 × 10^−5^ for 270°, approximately 41% change. Importantly, this difference in $\:\beta\:$ reveals that microstructural features, such as microcracks or mineral distribution, are highly direction-dependent, as $\:\beta\:$ significantly changes with the wave incident direction, even along the same axis. As a result, it can be concluded that $\:\beta\:$ provides more detailed evidence of the anisotropic nature in granite, particularly along cleavage planes, as shown in Fig. 11d.

## Conclusions

This study introduces an integrated ultrasonic platform designed to measure three independent acoustic properties – wave velocity, attenuation, and acoustic nonlinearity parameter – to elucidate the anisotropic nature of granite. The proposed platform combines traditional wave velocity and attenuation techniques with the longitudinal wave-based SHG technique, capable of quantifying microcracks inherently developed in three orthogonally located cleavage planes of two different granitic samples. Although wave velocity is useful for identifying differences in mechanical properties along the cleavage planes, precise classification of the cleavage planes could not be achieved due to its limited detection sensitivity. Moreover, the velocity measurements showed negligible difference between the two specimens, indicating its inadequacy in detecting cleavage-induced anisotropy. In contrast, the attenuation coefficient showed greater sensitivity than velocity in identifying the presence of anisotropy along the cleavage planes, with a slight difference observed between the two samples. Nevertheless, interpreting the anisotropic nature using the attenuation coefficient proved to be less informative at the microscale level. Importantly, the acoustic nonlinearity parameter exhibited clear superiority over other ultrasonic parameters in identifying the types of cleavage planes and differentiating the anisotropy of different granitic samples. This achievement is mainly attributed to the ability of the nonlinearity parameter to detect microstructural changes with high sensitivity. This study confirms the nonlinearity parameter as a novel and effective indicator capable of characterizing subtle features in granite, especially at the microscale. Implementing this nonlinearity parameter to characterize morphological features in granites holds great promise for achieving precise microscale characterization of various rock materials.

## Data Availability

The datasets used and analyzed during the current study are available from the corresponding author upon reasonable request.
